# Changes in cerebral oximetry during peritoneal insufflation for laparoscopic procedures

**DOI:** 10.4103/0972-9941.26651

**Published:** 2006-06

**Authors:** C L Gipson, G A Johnson, R Fisher, A Stewart, G Giles, J O Johnson, J D Tobias

**Affiliations:** Department of Anesthesiology, University of Missouri, Columbia, Missouri; * Department of Pediatrics, University of Missouri, Columbia, Missouri

**Keywords:** Cerebral oxygenation, laparoscopy, near infrared spectroscopy

## Abstract

**Background:**

Changes in cardiac output may occur during insufflation for laparoscopic procedures. However, there are limited data regarding its potential effects on cerebral oxygenation.

**Materials and Methods:**

Cerebral oxygenation (ScO_2_), end tidal CO_2_, heart rate, blood pressure and oxygen saturation by pulse oximetry were recorded every 5 minutes prior to insufflation, during insufflation and after desufflation. Minute ventilation was increased to maintain normocapnia and the depth of anesthesia was adjusted or fluids/phenylephrine administered to maintain the blood pressure within 20% of the baseline.

**Results:**

The cohort for the study included 70 adults for laparoscopic herniorrhaphy, gastric bypass or cholecystectomy. A total of 1004 ScO_2_ values were obtained during laparoscopy. The ScO_2_ decreased from the baseline in 758 of the 1004 data points. The ScO_2_ was 0–9 less than the baseline in 47.8% of the values, 10–19 less than the baseline in 24.9% of the values and 20–29 less than the baseline in 26 values (2.6%). Eighty-two (8.2%) of the values were less than 80% of the baseline value, while 25 values (2.5%) were less than 75% of the baseline value. Twelve patients had at least one ScO_2_ value that was less than 80% of the baseline and 6 had at least one ScO_2_ value that was less than 75% of the baseline. Four patients of the cohort had ScO_2_ values less than 80% of the baseline for more than 50% of the laparoscopic procedure.

**Conclusions:**

Although relatively uncommon, significant changes in cerebral oxygenation do occur in some patients during insufflation for laparoscopic surgery.

Laparoscopic surgery has become a popular surgical tool due to its less invasive nature, thereby providing a more rapid recovery and improved cosmetic outcome when compared to traditional open surgery. However, a requirement of the procedure is the insufflation of the peritoneal cavity with an inert gas, most commonly carbon dioxide (CO_2_). Although CO_2_ is generally accepted as the optimal gas for insufflation, increases in PaCO_2_ and resultant alterations in cerebral blood flow may occur.[1–3] Additionally, peritoneal insufflation and the increase in intra-abdominal pressure (IAP) result in increased systemic vascular resistance and decreased cardiac output.[1–6] These alterations in systemic hemodynamics may result in alterations in end-organ blood flow and oxygen delivery, especially in patients with co-morbid cardiovascular disease. To date, there are limited data regarding alterations in cerebral oxygenation during laparoscopic procedures. The current study uses near infrared spectroscopy (NIRS) to evaluate changes in cerebral oxygenation during laparoscopic procedures, in a cohort of adult patients.

## MATERIALS AND METHODS

This study was approved by the Institutional Review Board of the University of Missouri and informed consent was obtained from the patients. Patients scheduled for laparoscopic procedures of the abdomen requiring peritoneal insufflation were considered eligible for inclusion. The choice of anesthetic agents was not standardized and was at the discretion of the attending anesthesiologist. Cerebral oxygen saturation was continuously monitored using near infrared spectroscopy (INVOS 3100A, Somanetics Corporation, Troy, MI). After the induction of anesthesia, a single NIRS sensor was placed on the right side of the patient's forehead, with the caudad border approximately 1 cm above the eyebrow, with the medial edge at the midline according to the manufacturer's guidelines. Cerebral oxygen saturation (ScO_2_), end tidal CO_2_ (ETCO_2_), heart rate (HR), blood pressure (BP) and oxygen saturation by pulse oximetry (SaO_2_) were recorded every five minutes prior to insufflation, during insufflation and after desufflation. Peritoneal insufflation pressures were maintained at 8–12 mmHg. The surgical procedures were performed with the patient in the supine or at a 20–30° head-up position. During insufflation, minute ventilation was increased as needed, to maintain normocarbia. The blood pressure was maintained within 20% of baseline values by either increasing the concentration of the inhalational agent for increases in BP or the administration of a fluid bolus and/or phenylephrine for decreases in BP. The ScO_2_ values obtained prior to insufflation were averaged for each patient and used as that patient's baseline rSO_2_ for subsequent evaluations. The values obtained during insufflation were then compared to the baseline values and categorized as an increase in ScO_2_ from the baseline or as an absolute decrease in rSO_2_ of 0–9, 10–19, 20–29 or ≤ 30 or greater, from baseline. The number of values representing an ScO_2_ decrease to less than 75% and less than 80% of the baseline, were also determined. Patients who had any ScO_2_ value less than 80% of baseline, were compared to those without cerebral desaturation using a non-paired t-test and a contingency table, with a Fisher's exact test. All data are presented as the mean ± SD.

## RESULTS

The cohort for the study included 70 adult patients ranging in age from 27 to 80 years (50.3 ± 13.9 years) and in weight from 40 to 186 kgs (104.9 ± 31.6 kgs). Twelve patients were ASA II and 58 were ASA III. The surgical procedures included laparoscopic herniorrhaphy or cholecystectomy in 34 patients and laparoscopic gastric bypass in 36 patients. The anesthetic technique included premedication with midazolam (1–2 mg), followed by intravenous induction with thiopental or propofol. Maintenance anesthesia consisted of 1–1.5 MAC (minimum alveolar concentration) of a potent inhalational anesthetic agent, fentanyl (3–5 μg/kg) and neuromuscular blockade with intermittent doses of either cis-atracurium or rocuronium. The duration of insufflation and laparoscopy varied from 25 to 175 minutes (71.7 ± 33.3 minutes).

The baseline ScO_2_ varied from 47 to 95 (78 ± 11). A total of 1004 ScO_2_ values were obtained during laparoscopy. The ScO_2_ increased from the baseline during 246 of the 1004 data points and decreased from the baseline in 758 of the1004 data points. For the 758 of the 1004 ScO_2_ values that decreased from baseline during insufflation, the ScO_2_ decrease was 0–9 less than the baseline in 480 values (47.8% of all values), 10–19 less than the baseline in 252 values (24.9%) and 20–29 less than the baseline in 26 values (2.6%) [Table 1]. No ScO_2_ value was greater than 30 less than the baseline value. Eighty-two (8.2%) of the values were less than 80% of the baseline value, while 25 values (2.5%) were less than 75% of the baseline value. Twelve of the 70 patients (17%) had at least one ScO_2_ value that was less than 80% of the baseline value and 6 (0.09%) had at least one ScO_2_ value that was less than 75% of the baseline value. Four of the 70 patients of the cohort had ScO_2_ values less than 80% of the baseline, for more than 50% of the laparoscopic procedure. The demographics of patients with an ScO_2_ value less than 80% of the baseline during insufflation compared to patients without cerebral oxygen desaturation, are outlined in Table 2. There was no difference in the age of these patients (53.1 ± 10.8 years vs. 49.7 ± 14.5 years) or their ASA classification (ASA II and III: 0 and 13 vs. 12 and 45, *P* =0.1). However, these patients weighed more (120.2 ± 222. kgs vs. 101.4 ± 32.0 kgs, *P* =0.053), had longer durations of insufflation (67.5 ± 33.0 minutes vs. 90.4 ± 28.6 minutes, *P* =0.02) and were more likely to be undergoing a laparoscopic gastric bypass than a laparoscopic herniorrhaphy or cholecystectomy.

**Table 1:** Changes in cerebral oxygenation during laparoscopy

Absolute change in ScO_2_ from baseline	Number of values	Percentage of total
More than baseline	246	24.5
0–9 less	480	47.8
10–19	252	24.9
20–9	26	2.6
>30	0	0
= 80% of baseline	897	89.3
< 80% of baseline	82	8.2
< 75% of baseline	25	2.5

**Table 2:** Demographics of patients with ScO_2_ < 80% of baseline

	ScO_2_ < 80% of baseline	ScO_2_ ≤ 80% of baseline	*P* value
Age (years)	53.1 ± 10.8	49.7 ± 14.5	NS
ASA II and III	0 and 12	12 and 46	0.1
Weight (kgs)	120.2 ± 22.2	101.4 ± 32.0	0.053
Duration of laparoscopy (minutes)	90.4 ± 28.6	67.5 ± 33.0	0.02
Type of procedure:	10 and 2	26 and 32	0.06
Gastric bypass and cholescytectomy or herniorrphaphy			

## DISCUSSION

The potential use of NIRS, otherwise known as cerebral oximetry to monitor cerebral oxygenatio was first suggested by Jobsis in 1977.[7] NIRS is a non-invasive device that uses infrared light, a technique similar to pulse oximetry, to penetrate living tissues and estimate brain tissue oxygenation by measuring the absorption of infrared light by tissue chromophores (hemoglobin and cytochrome aa_3_).[8] Unlike standard pulse oximetry, pulsatile flow is not required and therefore the device works during CPB and other non-pulsatile states. Once the infrared light penetrates living tissue, the relative absorption of the infrared light at different wavelengths is dependent on the concentration of the various hemoglobin species (unoxygenated vs. oxygenated). Based on the relative absorption of the infrared light at various wavelengths, the specific concentration of the hemoglobin species can be determined using a modification of the Beer-Lambert's law and a determination of the relative degree of cerebral oxygenation can be made.[8–11] Unlike other methods for monitoring cerebral oxygenation, which are typically invasive, require prolonged periods of equilibration or involve ionizing radiation, NIRS is an inexpensive and noninvasive technique that may be used at the bedside.[12–14]

The ability of NIRS to detect sudden changes in cerebral oxygenation when cerebral blood flow is altered by extreme maneuvers, has been demonstrated by Levy *et al*[15] NIRS has also been shown to detect cerebral ischemia during less severe changes in cerebral oxygenation at frequencies similar to transcranial doppler velocimetry, thus providing important measurements of rapid and small changes in cerebral oxygenation.[12,16] Additional validation studies have been performed, demonstrating the accuracy of NIRS monitoring of brain oxygenation during conditions of hypoxemia, hypercapnia and hypocapnia in awake volunteers.[17,18] Pollard *et al* studied the response of the INVOS 3100A to arterial hypoxemia, induced by breathing a hypoxic mixture in 22 healthy adults and demonstrated that the cerebral oximeter reading correlated well with the calculated cerebral saturation.[18]

Clinical and biochemical studies have validated the correlation of NIRS monitoring with eventual neurologic outcome. Although the majority of the literature using NIRS has compared periods of cerebral desaturation with outcome assessed by clinical assessment of neurologic status, Plachky *et al*. compared changes in cerebral oxygenation using NIRS monitoring with neurologic damage assessed with biochemical markers, including neuron specific enolase (NSE) and S-100, a protein released during neuronal damage.[19] The cohort for the study included 16 adult patients undergoing orthotopic hepatic transplantation. NSE and S-100 were measured preoperatively and then again 24 hours after reperfusion of the donor liver. They noted that the decrease in ScO_2_ showed a significant correlation with the postoperative increase in NSE (r^2^ = 0.57) and S-100 (r^2^ = 0.52).

More recently, Casati *et al* have demonstrated that monitoring and acting on decreased ScO_2_ values may actually decrease the incidence of postoperative neurocognitive dysfunction in elderly patients undergoing major abdominal surgery under general anesthesia.[20] The 122 patients were randomly assigned to a treatment group in which the ScO_2_ value was monitored and maintained at ≥ 75% of preinduction values or a monitor only group, in which cerebral oximetry was monitored, but not visible to the anesthesiologist. Cerebral oxygen desaturation (≤ 75% of baseline) was observed in 11 patients of the treatment group (20%) and 15 patients in the control group (23%). The treatment protocol to treat cerebral oxygen desaturation, included ensuring adequate ventilation, checking head positioning, increasing the delivered oxygen concentration, allowing the arterial CO_2_ to increase and increasing the BP with fluids or vasoconstricting medications. Postoperative neurocognitive decline was evaluated using a mini-mental status examination (MMSE). Control patients with cerebral oxygen desaturation had a lower MMSE on postoperative day 7, longer PACU stays (median time of 47 minutes) and longer hospital stays (median of 24 days), when compared with patients in the treatment group whose cerebral oxygen desaturation was reversed by interventions. The latter group had a median PACU discharge time of 25 minutes (*P* =0.01) and a median hospital stay of 10 days (*P* =0.007).

In the current study, we noted that the majority of patients experienced a decrease in cerebral oxygenation during insufflation. Although the modest decreases are unlikely to be clinically significant, if we use the criteria of Casati *et al*,[20] who suggested that an ScO_2_ decrease to less than 75% of baseline correlates with neurocognitive changes assessed using the MMSE, 6 or approximately 1% of our patients would be at risk. Four of our patients had ScO_2_ values less than 80% of their baseline during more than 50% of the insufflation time. Patients who experienced an ScO_2_ value less than 80% of the baseline, weighed more and had a longer duration of peritoneal insufflation. As the majority of the patients were ASA III, aside from body weight, we cannot comment as to whether other co-morbid features increased the likelihood of cerebral oxygen desaturation.

Previous studies have evaluated changes in cerebral oxygenation using NIRS during insufflation for laparoscopy. Kitajima *et al* evaluated changes in cerebral oxygenation during laparoscopy using another type of cerebral oxygenation monitor (NIRO-500, Hamamatsu Photonics KK, Japan), in a study that included 12 adult patients (ASA I or II) undergoing laparoscopic cholecystectomy with a pneumoperitoneum of 10–12 mmHg.[21] Minute ventilation was not increased during laparoscopy. ETCO_2_ increased from a baseline of 33.9 ± 1.3 to a maximum of 52.8 ± 3.3 mmHg during laparoscopy. Both HbO_2_ (cerebral oxy-hemoglobin) and HbR (reduced hemoglobin) increased from the baseline following insufflation, which they related to changes in cerebral blood volume induced by the hypercapnia. No change in the oxygenation status of cytochrome aa_3_ was noted. The maximum increase of HbO_2_ (7.3 ± 2.8 μmol/L) occurred 90 minutes after the start of laparoscopy.

A follow-up study by the same group of investigators used the same technology to evaluate changes in cerebral oxygenation and cerebral blood volume during laparoscopic cholecystectomy in 12 adult ASA I and II patients, in whom minute ventilation was increased to maintain normocapnia.[22] They noted a small decrease in HbO_2_ from the baseline with insufflation (−1.4 ± 1.4 from baseline), with a more significant effect, 30 minutes after insufflation and assumption of a head-up positioning for the surgery (−5.1 ± 1.9 from baseline). No change in cytochrome aa_3_ was noted during the study.

Using the INVOS cerebral oximeter, de Waal *et al* evaluated changes in cerebral oxygenation and cerebral blood volume in 15 ASA I-III children during laparoscopic fundoplication, with an insufflation pressure of 5 to 8 mmHg.[23] During insufflation, the ETCO_2_ increased from 30.0 ± 2.8 to 38.3 ± 5.1 mmHg and PaCO_2_ increased from 32.0 ± 4.7 to 40.4 ± 5.9 mmHg. Cerebral oxygenation increased by 15.7 ± 8.8% and cerebral blood volume increased by 4.6 ± 8.8%.

The data in our current study are somewhat different that what has been reported in the 3 previous studies that have been mentioned.[21–23] We would postulate that several factors may account for this. We studied a much larger cohort of patients than have been previously studied and given the relatively low incidence of decreases in ScO_2_ values that we noted, it is possible that smaller studies including only 10–15 patients may miss such changes. Additionally, we collected ScO_2_ values at 5 minute intervals with 1000 values more, as compared with the other studies where cerebral oxygenation was assessed at 30 minutes intervals. We did not average our values, whereby a low value might be lost when averaged with other values, rather we compared each ScO_2_ value to the baseline, to assess its relative change. Our cohort mostly included ASA III patients, as opposed to the ASA I-II patients of Kitajima *et al*. Minute ventilation was increased to maintain normocapnia. As demonstrated by the studies of Kitajima *et al*,[21,22] this may affect HbO_2_ values, although the greatest difference they noted was when assuming the head-up position for the surgical procedure.

The current study did not separate randomized patients into treatment and non-treatment groups as was done by Casati *et al* and therefore we cannot comment as to whether treatment of decreases in ScO_2_ values would have an impact on the outcome. Recommendations for the treatment of decreases in ScO_2_ values include checking ventilator function and head positioning, increasing the F_i_O_2_, increasing the PaCO_2_ for values less than 35 mmHg, increasing mean arterial pressure with fluid or vasoconstrictors, followed by decreasing the cerebral metabolic rate of oxygen by the administration of propofol or a barbiturate if the above measures fail.

In summary, we noted that although most patients tolerated insufflation without significant effects on cerebral oxygenation, a subset of patients developed significant decreases in ScO_2_, which have been shown to correlate with neurocognitive changes in other studies. The limitations of the current study are, that we did not evaluate our patients postoperatively to see if changes in ScO_2_ correlated with postoperative neurocognitive changes and therefore we cannot determine if monitoring and treating changes in ScO_2_ can prevent postoperative neurocognitive changes, as has been previously demonstrated by the study of Casati *et al*.[20] As all the patients received a general anesthetic with a potent inhaled agent, no comment can be made on the impact of the anesthetic technique (potent inhaled agent vs. intravenous anesthesia). As we had a limited number of patients with what may be considered as clinically significant decreases in ScO_2_ values, an analysis to determine additional “at risk” features other than body weight and duration of insufflation, such as co-morbid features or intraoperative conditions (insufflation pressure, patient positioning and anesthetic technique), was not feasible. However, given that the deleterious physiologic effects of increased intraabdominal pressure during insufflation on stroke volume, cardiac output and other hemodynamic variables have been well documented,[24] it is likely that alterations in cerebral oxygenation may occur. Therefore, non-invasive monitoring of cerebral oxygenation appears to be warranted in this population, especially in patients with associated co-morbid conditions.
